# Collaborative variable neighborhood search for multi-objective distributed scheduling in two-stage hybrid flow shop with sequence-dependent setup times

**DOI:** 10.1038/s41598-022-19215-3

**Published:** 2022-09-20

**Authors:** Jingcao Cai, Shejie Lu, Jun Cheng, Lei Wang, Yin Gao, Tielong Tan

**Affiliations:** 1 School of Mechanical Engineering, Anhui Polytechnic University, Wuhu, 241000 People’s Republic of China; 2 Institute of Engineering and Technology, Hubei University of Science and Technology, Xianning, 437100 People’s Republic of China; 3 School of Computer Science and Artificial Intelligence, Wuhan Textile University, Wuhan, 430074 People’s Republic of China; 4 Wuhu Kepu Intelligent Equipment Co., Ltd, Wuhu, 241000 People’s Republic of China

**Keywords:** Mechanical engineering, Computer science

## Abstract

Distributed scheduling is seldom investigated in hybrid flow shops. In this study, distributed two-stage hybrid flow shop scheduling problem (DTHFSP) with sequence-dependent setup times is considered. A collaborative variable neighborhood search (CVNS) is proposed to simultaneously minimize total tardiness and makespan. DTHFSP is simplified by incorporating factory assignment into machine assignment of a prefixed stage, and its solution is newly represented with a machine assignment string and a scheduling string. CVNS consists of two cooperated variable neighborhood search (VNS) algorithms, and neighborhood structures and global search have collaborated in each VNS. Eight neighborhood structures and two global search operators are defined to produce new solutions. The current solution is periodically replaced with a member of the archive farthest from it. Experiments are conducted , and the computational results validate that CVNS has good advantages over the considered DTHFSP.

## Introduction

Hybrid flow shop scheduling problems (HFSP) are not uncommon in many real-life manufacturing industries such as electronics, paper, textile and semiconductor^1,2^. Two-stage HFSP as the particular case of the general HFSP is proved to be NP-hard by Gupta^3^ decades ago even in the special case that two machines exist in one stage and a single machine is allocated in another stage. Two-stage HFSP also has attracted much attention due to its practical and diverse applications.

Many methods, including heuristic, exact algorithm and meta-heuristic, have been applied to solve two-stage HFSP because of its high complexity. Allaoui and Artiba^4^ investigated two-stage HFSP with availability constraint by using an exact method and several heuristics to minimize makespan. Wang and Liu^5^ proposed a genetic algorithm (GA) for two-stage no-wait HFSP. Tan et al.^6^ presented a hybrid decomposition approach with variable neighborhood search (VNS) for the problem with batch processing machines. Yang^7^ developed some heuristics for the special cases of the problem with dedicated machines. Wang and Liu^8^ presented a heuristic-based on branch-and-bound for the problem with dedicated machines. Setup times extensively exist in real-life manufacturing situations^9^ and are also handled in two-stage HFSP. Lin and Liao^10^ applied a heuristic method to solve the problem with setup times and dedicated machines. Hekmatfar et al.^11^ presented some heuristics and a hybrid GA for two-stage reentrant HFSP with setup times. Lee et al.^12^ provided a hybrid method based on beam search and NEH for the problem with setup times. The previous works also considered batching and scheduling^13^, interval processing times^14^, assembly^15^, renewable resources^16^ and preventive maintenance^17^ and applied meta-heuristics such as tabu search^16,17^, artificial bee colony^18^ and imperialist competitive algorithm^19^ to solve two-stage HFSP.

The above works are often implemented in a single factory. To our knowledge, two-stage HFSP is not investigated in multi-factory environments. Production has shifted from a single factory to a multi-factory production network with the development of globalization. In a multi-factory production network, each factory can be considered as an individual entity with different location and efficiency and different constraints such as worker cost, tax, close to suppliers, etc. The distributed scheduling problems in multi-factory networks reveal new features such as many sub-problems and strong coupled relations among sub-problems. In recent years, distributed scheduling in multi-factory networks has attracted some attention. A number of results have been obtained on distributed scheduling in single machine^20^, parallel machines^21–26^, flow shop^27–43^ and hybrid flow shop^44–65^ etc.

Flow shop scheduling is a special case of HFSP. In recent years, distributed flow shop scheduling problems (DFSP) had been considered, and several results have been obtained. Distributed permutation flow shop scheduling problem is often dealt with. For the problem with makespan minimization, Lin et al.^27^ proposed a modified iterated greedy (IG) algorithm, Naderi and Ruiz^28^ applied a scatter search algorithm, Xu et al.^29^ presented a hybrid immune algorithm, Wang et al.^30^ designed an effective estimation of distribution algorithm and Gao et al.^31^ developed an efficient tabu search. DFSP with practical constraints such as blocking^32^, no-wait^33,34^ and assembly^35–37^ is often addressed. For DFSP with sequence-dependent setup times (SDST), Hatami et al.^40^ proposed two heuristics, a VNS and an IG. DFSP with multiple objectives is also studied^41,42^.

Distributed HFSP has also been considered. For this problem with the optimization objective of minimizing maximum completion time, the common methods are iterative greed algorithm^44,60^, shuffled frog leaping algorithm^47^, hybrid brain storm optimization algorithm^49,54^, artificial bee colony algorithm^55–57^, and cooperative memetic algorithm^62^ etc. For the problem with multi-objective optimization, the methods used are genetic algorithm^59^, variable neighborhood search^48^, shuffled frog leaping algorithm^45,46,51,64^, teaching-learning-based optimization^52^, cooperative coevolution algorithm^63,65^, decomposition-based multi-objective optimization^50^, iterated greedy algorithm^61^ and other evolutionary algorithms^53^.

As stated above, the literature on DFSP is mainly about optimizing a single objective such as makespan by using GA and IG, etc. DFSP with multiple objectives and DFSP with SDST is not considered fully; on the other hand, two-stage HFSP is investigated fully in the past decade; however, two-stage HFSP are hardly handled in the multi-factory production network, let alone distributed two-stage hybrid flow shop scheduling problem (DTHFSP) with SDST and objectives such as makespan and total tardiness. Because of the diverse applications of two-stage HFSP in real-life manufacturing situations, it is necessary to address bi-objective DTHFSP with constraints such as SDST.

In general, DTHFSP composes three sub-problems: factory assignment, which assigns jobs to an appropriate factory; machine assignment, which allocates each job to a machine in its assigned factory and scheduling one deciding the sequence of jobs on each machine. When each job is directly allocated to a machine at a predetermined stage *s* and the results of factory assignment can be obtained directly, that is, factory assignment can be incorporated into machine assignment of a predetermined stage; as a result, the number of sub-problems diminishes and the problem is simplified.

VNS^44^ is a local-search-based meta-heuristic, which explores increasingly several neighborhoods of the current solution and jumps from this solution to a new one if and only if an improvement has been made. In this way, favorable features of the current solution will be kept and used to obtain promising neighborhood solutions. VNS has been applied to various production scheduling problems^6,26,35,66–70^; however, VNS is just hybridized with other methods and seldom applied independently^35^ to solve two-stage HFSP and DFSP.

In this study, DTHFSP with SDST is considered, and a collaborative variable neighborhood search (CVNS) is proposed to simultaneously minimize total tardiness and makespan. The main characteristics of CVNS are as follows. DTHFSP is simplified by incorporating factory assignment into machine assignment of a prefixed stage, and a new coding is adopted by representing a solution with a machine assignment string and a scheduling string. CVNS comprises two cooperated VNS, and neighborhood structures and global search have collaborated in each VNS. Eight neighborhood structures and two global search operators are defined to produce new solutions. The current solution is periodically replaced with a member of the archive farthest from it. Several experiments are conducted on many instances, and CVNS is compared with two famous multi-objective GAs and a multi-objective tabu search algorithm. The computational results demonstrate that CVNS has good advantages over the considered DTHFSP.

The remainder of the paper is organized as follows. The problem under study is described in “Problem descriptions” and followed by the introduction to VNS in “CVNS for DTHFSP with SDST”. CVNS for bi-objective DTHFSP is described in “Computational experiments”. Numerical test experiments on CVNS are reported in “Conclusions”, the conclusions are summarized in the final section, and some topics for future research are provided.

## Problem descriptions

Bi-objective DTHFSP is depicted as follows. There are *n* jobs distributed among *F* factories at different sites. Each factory $Fac_f$ has a two-stage hybrid flow shop, where there are $m_f$ identical parallel machines at stage one and a single machine in stage two. All jobs are available at time zero. Machines $M_{1,s_f + 1} , \ldots ,M_{1,s_f + m_f }$ are located in the first stage of factory *f*, where $s_f = \sum \nolimits _{l = 1}^{f - 1} {m_l } ,f > 1$, $s_1=0$. There are total $W = \sum \nolimits _{f = 1}^{F} {m_f }$ parallel machines. $M_{2,f}$ indicates the single machine in the second stage of factory $Fac_f$. Each job $J_i$ has a due date $d_i$ and a processing time $p_{isf}$ at stage *s* of factory $Fac_f$. SDST is considered. $u_{jisf}$ indicates the setup time for job $J_i$ on each machine of stage *s* in factory $Fac_f$ when job $J_j$ is processed directly before it on the same machine. $u_{0isf}$ represents initial setup time of the first job $J_i$ processed on machine at stage *s* of factory $Fac_f$. In general, $u_{jisf_1}\ne u_{jisf_2}$ and $u_{0isf_1} \ne u_{0isf_2}$ for $f_1 \ne f_2$, and $u_{jisf}\ne u_{jigf}$ and $u_{0isf} \ne u_{0igf}$ for $s\ne g$.

DTHFSP has some constraints on jobs and machines.

Each machine can process at most one operation at a time,

No jobs may be processed on more than one machine at a time,

Operations cannot be interrupted,

All machines are available at all times etc.

**Figure 1:**
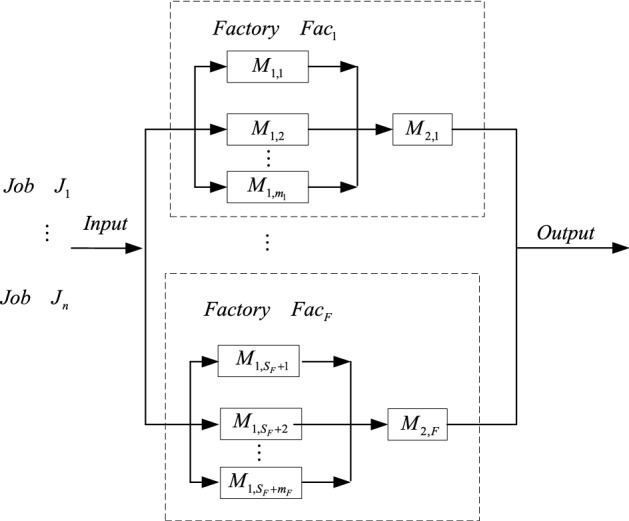
Multi-factory system of DTHFSP.

In general, DTHFSP can be categorized into three sub-problems, (1) factory assignment used to decide which jobs are allocated to each factory; (2) machine assignment; (3) scheduling. Machines in each factory are fixed in advance. If we allocate each job to a machine at the first stage and then decide the number of jobs on machines at the first stage of each factory, then we can find that jobs are allocated in each factory, in this way, two assignment problems are combined into machine assignment of the first stage, and DTHFSP is simplified, which consists of machine assignment of the first stage and scheduling. Two assignment sub-problems cannot be integrated into machine assignment of the second stage because machine assignment of the first stage must be done and three sub-problems still exist. Figure 1 shows a multi-factory system of the problem.

The goal of DTHFSP is to allocate jobs to machines from different factories and schedule jobs on each machine to minimize two objectives, the maximum completion time $C_{max}$ and the total tardiness $T_{tot}$. Table 1 provides the description of notations. The mathematical model of DTHFSP is as follows:

1 $$\begin{aligned}{C_{\max }} = \mathop {\max }\limits _{i \in \{ 1,2, \ldots ,n\} } \{ {C_i}\} \end{aligned}$$

2 $$\begin{aligned}T_{tot} = \sum \limits _{i = 1}^n {\max \left\{ {C_i - d_i ,0} \right\} } \end{aligned}$$

3 $$\begin{aligned}\sum \limits _{f = 1}^F {{X_{if}}} = 1,\forall i \end{aligned}$$

4 $$\begin{aligned}\sum \limits _{l = 1}^{{m_{f}}} {{Y_{ifsl}}} = {X_{if}},\forall i,f,s=1 \end{aligned}$$

5 $$\begin{aligned}Y_{if21}=X_{if}, \forall i,f \end{aligned}$$

6 $$\begin{aligned}s{t_{i1}} \ge 0,\forall i \end{aligned}$$

7 $$\begin{aligned}s{t_{i2}} \ge e{t_{i1}},\forall i \end{aligned}$$

8 $$\begin{aligned}e{t_{is}} = s{t_{is}} + \mu _{jisf}\times A_{jis}+\mu _{0isf}\times B_{is} + \sum \limits _{f = 1}^F {{\sum \limits _{l = 1}^{{m_{f}}} {({{{p_{isf}}}}} \times {X_{if}} \times {Y_{ifsl}})} } ,\forall i,s=1 \end{aligned}$$

9 $$\begin{aligned}e{t_{is}} = s{t_{is}} +\mu _{jisf}\times A_{jis}+\mu _{0isf}\times B_{is}+ \sum \limits _{f = 1}^F {{ {({{{p_{isf}}}}} \times {X_{if}} \times {Y_{ifs1}})} } ,\forall i,s=2 \end{aligned}$$

10 $$\begin{aligned}{Z_{ii'fs}} + {Z_{i'ifs}} \le 1,\forall f,s,i,i' \end{aligned}$$

11 $$\begin{aligned}{Z_{ii'fs}} + {Z_{i'ifs}} \ge {Y_{ifsl}} + {Y_{i'fsl}} - 1,\forall f,s,i', i \end{aligned}$$

12 $$\begin{aligned}{s{t_{i's}} \ge e{t_{is}} - U \times (3 - {Y_{ifsl}} - {Y_{i'fsl}} - {Z_{ii'fs}}),} {\forall i \ne i',f,s,l } \end{aligned}$$

13 $$\begin{aligned}{X_{if}} \in \{ 0,1\} ,\forall i,f \end{aligned}$$

14 $$\begin{aligned}{Y_{ifsl}} \in \{ 0,1\} ,\forall i,f,s,l \end{aligned}$$

15 $$\begin{aligned}{Z_{ii'fs}} \in \{ 0,1\} ,\forall i,i',f,s \end{aligned}$$

where Eq. (1) is to minimize maximum completion time; Eq. (2) is to minimize total tardiness; constraint (3) describes that each job can only be processed in one factory; constraints (4–5) show that a job can only be assigned to one machine at each stage in each factory; constraint (6) denotes that each job can be processed after zero time; constraint (7) indicates that jobs can be processed in the second stage only after it is processed in the first stage; constraints (8–9) shows that the process cannot be interrupted; constraints (10–12) demonstrates that each machine can only process one job at one time; constraints (13–15) are binary decision variables.

**Table 1:** Notations and descriptions.

Notations	Descriptions
$C_i$	The completion of job $J_i$
$st_{is}$	The process start time of $J_i$ at stage *s*
$et_{is}$	The process end time of $J_i$ at stage *s*
$X_{if}$	If $J_i$ is processed in $Fac_f$, $X_{if}=1$, otherwise $X_{if}=0$
$Y_{ifsl}$	If $J_i$ is processed in the *l*-th machine at stage *s* in factory *f*, $Y_{ifsl}=1$, otherwise $Y_{ifsl}=0$
$Z_{ii'fs}$	If $J_i$ is processed before $J_{i'}$ at stage *s* in factory *f*, $Z_{ii'fs}=1$, otherwise $Z_{ii'fs}=0$
$A_{jis}$	If job $J_i$ is processed directly before $J_j$ on the same machine at stage *s*, $A_{jis}=1$, otherwise $A_{jis}=0$
$B_{is}$	If job $J_i$ is the first job processed on a machine at stage *s*, $B_{is}=1$, otherwise $B_{is}=0$

## CVNS for DTHFSP with SDST

DTHFSP is regarded as the problem with machine assignment of the first stage, and scheduling and a two-string representation is adopted. Then eight neighborhood structures and two global search operators are used to produce new solutions; finally, a CVNS is developed based on the cooperation of two VNS algorithms and the collaboration of global search and neighborhood search.

### Introduction to VNS

The steps of basic VNS are presented as follows^44^.

- Step 1: Initialization. Select the set of neighborhood structures ${{\mathscr {N}}}_k$($k = 1,2, \ldots ,k_{max}$), produce an initial solution *x*, choose a stopping condition.
- Step 2: Repeat the following steps until the stopping condition is met.
  1. $$k=1$$.
  2. repeat the following steps until $k=k_{max}$.
    - Randomly generate a solution $x^{'} \in {\mathscr {N}}_k \left( x \right)$.
    - Let $$x^{'}$$ as the initial solution, apply local search methods to it and obtain local optimum $$x^{''} $$.
    - If the local optimum $$x^{''} $$ is better than the incumbent, replace *x* with $$x^{''}$$ and continue the search with $${{\mathscr {N}}}_1$$; otherwise $$k=k+1$$.

where ${{\mathscr {N}}}_k$ is the neighborhood structure and ${{\mathscr {N}}}_k \left( x \right)$ indicates the neighborhood of *x* obtained by using ${{\mathscr {N}}}_k$.

The stopping condition may be the maximum CPU time allowed, the maximum number of iterations, etc.

### Two-string representation

In this study, a two-string representation is applied to represent the solution of DTHFSP. For the problem with *n* jobs and *W* parallel machines, a solution is denoted by a machine assignment string $[M_{1,h_1},M_{1,h_2},\ldots , M_{1,h_n}]$ and a scheduling string $[q_1,q_2,\ldots , q_n]$. Machine $M_{1,h_i}$ is allocated for job $J_i$ on the first stage, $h_i\in [1,W]$ and $q_l$ corresponds to $J_l$. The scheduling string is a random key one, suppose that jobs $J_i,J_{i+1},\ldots ,J_j$ are processed on the same machine of the first stage, that is, $M_{1,h_i}=M_{1,h_{i+1}},\ldots , =M_{1,h_j}$, the processing sequence of these jobs on the machine of the first stage is decided by the ascending order of $q_l, l\in [i,j],i<j$. The processing sequence of all jobs in a factory on the single machine of the second stage is also determined by the ascending order of their $q_l$.

The decoding procedure is described below. Machine assignment of each job in the first stage is first done according to the first string; then in each factory *f*, the allocated jobs of each parallel machine are processed sequentially in terms of the ascending order of their $q_l$, the sequence of the assigned jobs of factory *f* is decided on machine $M_{2,f}$ according to the ascending order of their $q_l$. Each assigned job is processed sequentially after its processing at the first stage is finished.

**Figure 2:**
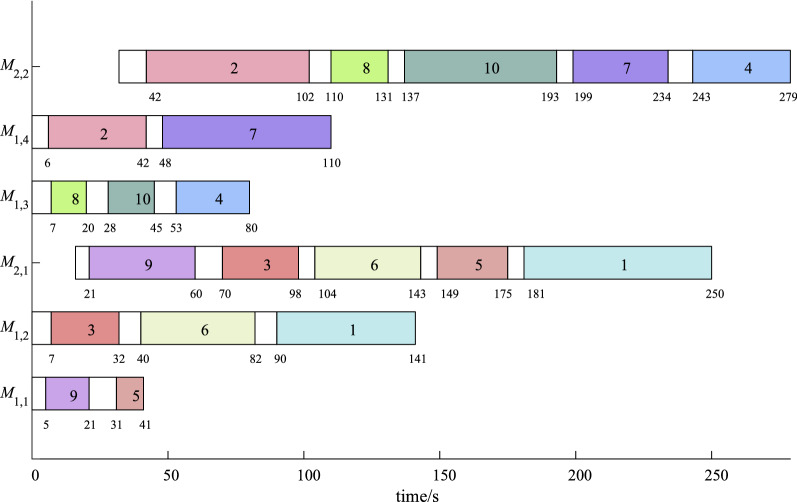
The schedule of the possible solution.

For a example with two factories, 10 jobs, $M_{1,1}, M_{1,2}, M_{2,1}$ and $M_{1,3}, M_{1,4}, M_{2,2}$, a possible solution is $[M_{1,2},M_{1,4},M_{1,2},M_{1,3},$ $M_{1,1},M_{1,2},M_{1,4},M_{1,3},M_{1,1},M_{1,3}]$ and [0.55, 0.32, 0.18, 0.95, 0.44, 0.39, 0.87, 0.56, 0.05, 0.75]. Jobs $J_1,J_3,J_6$ are assigned on machine $M_{1,2}$, $q_1=0.55,q_3=0.18,q_6=0.39$, so the sequence is $J_3,J_6,J_1$. We also can be found that $J_1,J_3,J_5,J_6,J_9$ are allocated in factory 1 and other jobs are processed in factory 2. The processing sequence on single machine of factory 1 is $J_9,J_3,J_6,J_5,J_1$. Figure 2 shows a schedule of the solution.

### Neighborhood structures

Eight neighborhood structures are defined for the current solution *x*, four for machine assignment of the first stage and four for scheduling.

Neighborhood structure ${{\mathscr {N}}}_1$ is executed in a factory and described below. A factory *f* is randomly chosen, and then a parallel machine $M_{1,l}$ with maximum workload and $M_{1,g}$ with minimum workload are decided at the first stage of factory *f*, a job on $M_{1,l}$ is finally moved to machine $M_{1,g}$, where the workload of a machine is the sum of setup time and processing time of all jobs processed on this machine. For the solution in Fig. 2, factory 1 is chosen, $M_{1,2}$ and $M_{1,1}$ have the maximum workload and minimum workload,respectively, finally, job 1 is moved from $M_{1,2}$ to $M_{1,1}$.

Neighborhood structure ${{\mathscr {N}}}_2$ is performed between two factories and shown as follows. Two factories $f_1,f_2,f_1 \ne f_2$ are stochastically selected; then in each factory, a parallel machine $M_{1,l_i}$ with maximum workload and a machine $M_{1,g_i}$ with minimum workload are respectively decided, $i=1,2$; finally, if the workload of $M_{1,l_1}$ is greater than that of $M_{1,l_2}$, then move a job from $M_{1,l_1}$ to $M_{1,g_2}$, otherwise allocate a job from $M_{1,l_2}$ to $M_{1,g_1}$. For factories 1 and 2 in Fig. 2, $M_{1,2}$ and $M_{1,1}$ have the maximum and minimum workload, respectively. $M_{1,4}$ and $M_{1,3}$ are machines in factory 2 with the maximum workload and minimum workload. Workload of $M_{1,2}$ is greater than that of $M_{1,4}$, job 3 is moved from $M_{1,2}$ to $M_{1,3}$.

Neighborhood structure ${{\mathscr {N}}}_3$ is also done between two factories. Two factories $f_1,f_2, f_1 \ne f_2$ are stochastically selected; then in each factory, a parallel machine $M_{1,l_i}$ with maximum workload and a parallel machine $M_{1,g_i}$ with minimum workload are respectively decided, $i=1,2$; finally, if the workload of $M_{1,l_1}$ is greater than that of $M_{1,l_2}$, then move a job from $M_{1,l_1}$ to $M_{1,g_2}$ and assign a job from $M_{1,g_2}$ to $M_{1,l_1}$, otherwise a job is moved from $M_{1,l_2}$ to $M_{1,g_1}$ and another job is from $M_{1,g_1}$ to $M_{1,l_2}$.

${{\mathscr {N}}}_4$ is applied to lead to a new machine assignment for the chosen jobs described below. Randomly choose a job $J_i$ from a factory *f* with the maximum completion time, that is, this maximum completion time is makespan, then a parallel machine $M_{1,w}$ of the first stage is stochastically chosen from the factory *g* with the minimum completion time and the job $J_i$ is assigned on the machine $M_{1,w}$. In Fig. 2, the maximum completion times of two factories are 279 and 250, job 4 is moved from $M_{1,3}$ to $M_{1,1}$.

${{\mathscr {N}}}_1,{{\mathscr {N}}}_2,{{\mathscr {N}}}_3,{{\mathscr {N}}}_4$ directly act on the schedule of a solution, and the machine assignment string can be obtained after they are done. These neighborhood structures are used to produce new assignments for some jobs at the first stage.

The scheduling string is used to decide the sequences of jobs on each parallel machine and single machine in each factory. Neighborhood structures ${{\mathscr {N}}}_5,{{\mathscr {N}}}_6$ are applied to result in some changes to the second string. ${{\mathscr {N}}}_5$ generates new solutions by swapping two pairs of randomly chosen genes $q_l$ and $q_g$, $l\ne g$. ${{\mathscr {N}}}_6$ produces new solutions by inserting a randomly selected gene $q_l$ into a new position $g,l\ne g$.

${{\mathscr {N}}}_7$ is depicted for each factory *f*: sort all jobs in factory *f* in the ascending order of due dates and then let the sequence of their $q_l$ of these jobs be identical with the order of due dates. In Fig. 2, for jobs $J_9,J_3,J_6,J_5,J_1$ in factory 2, suppose that their due dates are 35, 45, 65, 23, 78, jobs are sorted as $J_5,J_9,J_3,J_6,J_1$ and the sequence of their $q_l$ is $q_5,q_9,q_3,q_6,q_1$.

The steps of ${{\mathscr {N}}}_8$ are identical with ${{\mathscr {N}}}_7$ except that the ascending order of processing times on single machine substitutes for that of the due date. For the factory 2 in Fig. 2, suppose that processing times jobs of $J_9,J_3,J_6,J_5,J_1$ on single machine are 10, 13, 8, 5, 23, 9, these jobs are sorted as $J_6,J_3,J_1,J_9,J_5$ and the sequence of their $q_l$ is $q_6,q_3,q_1,q_9,q_5$, then the scheduling string becomes [0.39, 0.32, 0.18, 0.95, 0.55, 0.05, 0.87, 0.56, 0.44, 0.75].

**Figure 3:**
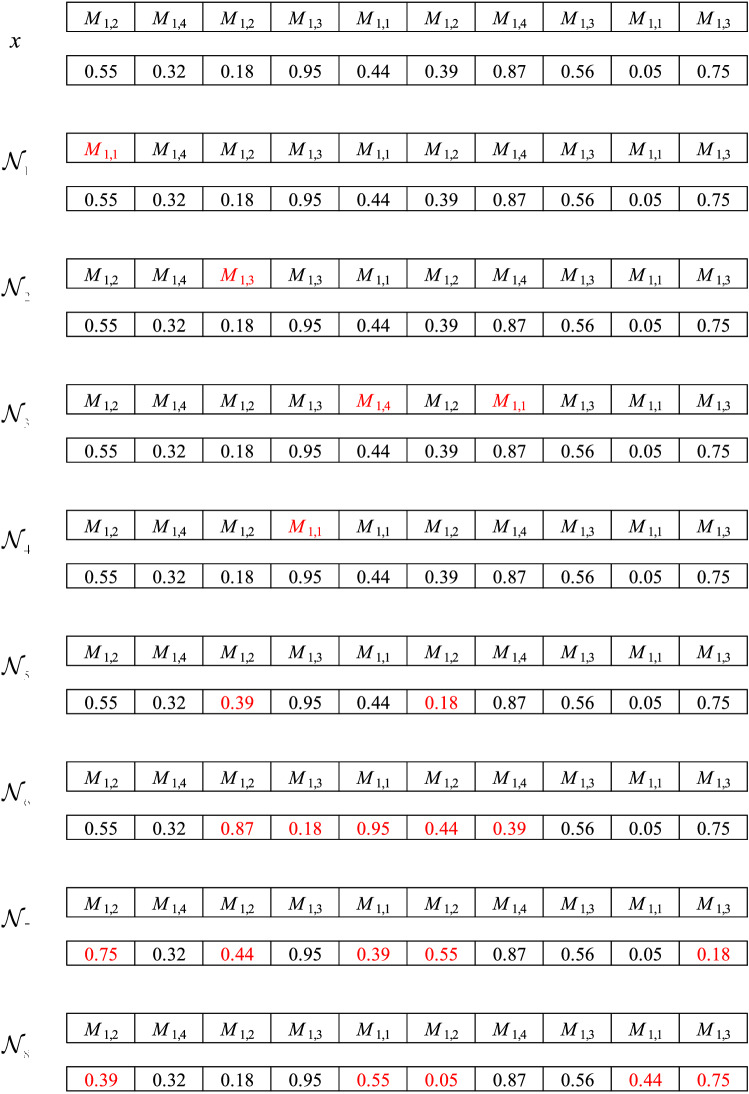
The explanation of 8 neighborhood structures.

Figure 3 explains 8 neighborhood structures.

### Global search

In general VNS, only neighborhood search is used to produce new solutions. To intensify the global search ability of VNS, two two-point global searches are adopted between the current solution *x* and a member *y* of archive $\Omega$, which is utilized to store non-dominated solutions. The first global search $GS_1$ acts on the machine assignment string of *x* and *y*. A member *y* is randomly chosen from $\Omega$, two positions $k_1,k_2$ are randomly selected and machines between $k_1$ and $k_2$ on the first string of *x* are directly replaced with those on the first string of *y*. The second global search $GS_2$ is executed in the same way on the scheduling string; genes of *x* between two positions are displaced by those of *y* on the same positions.

### Descriptions on CVNS

The detailed procedure of CVNS is described as follows.

1. Initialization. Randomly produce an initial solution *x* and build an initial archive $\Omega$, $t=1,\theta _1=\theta _2=1$.
2. If $\theta _1=1$, $x^{'} \in {{\mathscr {N}}}_1(x)$; if $\theta _1=2$, $x^{'} \in {{\mathscr {N}}}_5(x)$; if $\theta _1=3$, $x^{'} \in {\mathscr {N}}_3(x)$; if $\theta _1=4$ and a member $y\in \Omega$ and $y\ne x$ exists, $GS_1$ is executed between *x* and *y* and $x^{'}$ is gotten.
3. If solution $x^{'}$ is not dominated by *x*, then replace *x* with $x^{'}$, update $\Omega$ and $\theta _1=1$; otherwise $\theta _1=\theta _1+1$ and let $\theta _1=1$ if $\theta _1=5$.
4. $x^{'}\in {{\mathscr {N}}}_4(x)$ is generated, compare $x^{'}$ with *x* and update $\Omega$ as done in Step3.
5. If $\theta _2=1$, $x^{'} \in {{\mathscr {N}}}_5(x)$; if $\theta _2=2$, $x^{'} \in {{\mathscr {N}}}_6(x)$; if $\theta _2=3$, $x^{'} \in {mathscr{N}}_2(x)$; if $\theta _2=4$ and a member $y\in \Omega$ with $y\ne x$ exists, $GS_2$ is done between *x* and *y*, and $x^{'}$ is produced.
6. If solution $x^{'}$ is not dominated by *x*, then replace *x* with $x^{'}$,update $\Omega$ and $\theta _2=1$; otherwise $\theta _2=\theta _2+1$ and let $\theta _2=1$ if $\theta _2=5$.
7. ${{\mathscr {N}}}_7$ and ${{\mathscr {N}}}_8$ are sequentially performed on *x*, $x^{'}$ is generated, compare $x^{'}$ with *x* and update $\Omega$ as done in Step3.
8. $t=t+5$. If *t* is exactly divided by $\beta$, then a solution $y\in \Omega$ being farthest to *x* substitutes for *x*.
9. If algorithm running time exceeds $max\_it$, then go to step 2.
10. Output the archive $\Omega$.

where $max \_it$ indicates the maximum algorithm running time, $\beta$ is an integer, if $C_{max}(x^{'})\le C_{max}(x)$ and $T_{tot}(x^{'})\le T_{tot}(x)$, and $C_{max}(x^{'})< C_{max}(x)$ or $T_{tot}(x^{'})< T_{tot}(x)$, then $x^{'}$ dominates *x* or *x* is dominated by $x^{'}$. If there is no dominating relation between *x* and $x^{'}$, they are non-dominated by each other. That $x^{'}$ dominates *x* or is non-dominated with *x* means that $x^{'}$ is not dominated by *x*.

The archive $\Omega$ is used to store the non-dominated solutions obtained by CVNS. The set $\Omega$ is updated using the new solution $x^{'}$ in the following way: the new solution $x^{'}$ is added into $\Omega$, and all solutions of $\Omega$ are compared according to Pareto dominance and the dominated ones are removed from $\Omega$.

In CVNS, global search and neighborhood search have collaborated, two VNS algorithms have cooperated, some neighborhood structures are applied to improve solution quality, and the current solution is periodically replaced, these strategies can make a good balance between exploration and exploitation and keep high diversity, so CVNS can have promising performances.

## Computational experiments

Extensive experiments are conducted on a set of problems to test the performance of CVNS for bi-objective DTHFSP. All experiments are implemented by using Microsoft Visual C++ 2015 and run on a 4.0G RAM 2.00GHz CPU PC.

### Test instances, comparative algorithms and metrics

188 instances are randomly produced, and Table 2 describes the basic information for these instances. $u_{0isf},u_{jisf} \in [5,10]$ and $p_{isf}\in [1,100]$ in instances 1-94. $u_{0isf},u_{jisf} \in [5,10]$ and $p_{isf}\in [10,80]$ in instances 95-188. Cai et al.^45^ provided the method to generate due data $d_i$, which is defined by

16 $$\begin{aligned}{d_i} \in [{d_{\min }},{d_{\max }}], \end{aligned}$$

17 $$\begin{aligned}{d_{\min }} = (\mathop {\min }\limits _{i,s,f} {p_{isf}} + \mathop {\min }\limits _{j,i,s,f} {u_{jisf}}) \times 2, \end{aligned}$$

18 $$\begin{aligned}{d_{\max }} = \frac{{(\mathop {\max }\limits _{i,s,f} {p_{isf}} + \mathop {\max }\limits _{j,i,s,f} {u_{jisf}}) \times 2 \times n}}{{F \times \mathop {\max }\limits _f {m_f}}}. \end{aligned}$$

As shown in Table 2, instance 60 includes 4 factories, 150 jobs, and the first processing stage of the 4 factories contains 2, 2, 3 and 3 parallel machines, respectively. The processing time of each job is a uniformly distributed random number. An example for generating due dates is provided as follows. The processing time and setup time related to job $$J_3$$ are $$p_{3sf}$$ and $$u_{j3fs} $$. Assuming that $$\mathop {\min }\nolimits _{i,s,f} {p_{isf}}=20$$, $$\mathop {\min }\nolimits _{j,i,s,f} {u_{jisf}}=5$$, $$\mathop {\max }\nolimits _{i,s,f} {p_{isf}}=80$$ and $$\mathop {\max }\nolimits _{j,i,s,f} {u_{jisf}}=10$$, then $${d_i} \in [50,900]$$ according to Eqs. (16–18), so the value of $$d_3$$ could be 100.

**Table 2:** Basic information on instances.

Instance	*F*	$m_f$	Instance	*n*
1–10, 95–104	2	3, 3	1, 11, 21, 31, 41, 51, 61, 95, 105, 115, 125, 135, 145,155	30
11–20, 105–114	2	2, 4	2, 12, 22, 32, 42, 52, 62, 96, 106, 116, 126, 136, 146, 156	40
21–30, 115–124	3	3, 3, 3	3, 13, 23, 33, 43, 53, 63, 71, 79, 87, 97, 107, 117, 127, 137, 147, 157, 165, 173, 181	50
31–40, 125–134	3	2, 3, 4	4, 14, 24, 34, 44, 54, 64, 72, 80, 88, 98, 108, 118, 128, 138, 148, 158, 166, 174, 182	60
41–50, 135–144	4	2, 2, 2, 2	5, 15, 25, 35, 45, 55, 65, 73, 81, 89, 99, 109, 119, 129, 139, 149, 159, 167, 175, 183	70
51–60, 145–154	4	2, 2, 3, 3	6, 16, 26, 36, 46, 56, 66, 74, 82, 90, 100, 110, 120, 130, 140, 150, 160, 168, 176, 184	80
61–70, 155–164	4	2, 3, 4, 5	7, 17, 27, 37, 47, 57, 67, 75, 83, 91, 101, 111, 121, 131, 141, 151, 161, 169, 177, 185	90
71–78, 165–172	5	3, 3, 3, 3, 3	8, 18, 28, 38, 48, 58, 68, 76, 84, 92, 102, 112, 122, 132, 142, 152, 162, 170, 178, 186	100
79–86, 173–180	5	2, 3, 3, 3, 4	9, 19, 29, 39, 49, 59, 69, 77, 85, 93, 103, 113, 123, 133, 143, 153, 163, 171, 179, 187	120
87–94, 181–188	5	2, 3, 4, 5, 6	10, 20, 30, 40, 50, 60, 78, 86, 94, 104, 114, 124, 134, 144, 154, 172, 180, 188	150

As stated above, bi-objective DTHFSP is not considered, so there are no existing methods as comparative algorithms. To test the performance of CVNS, we choose three famous multi-objective evolutionary algorithms called non-dominated sorting genetic algorithm-II (NSGA-II^71^), strength Pareto evolutionary algorithm2 (SPEA2^72^) and multi-objective tabu search (MOTS^73^).

NSGA-II has a very competitive performance in solving the multi-objective problem. In NSGA-II, a non-dominated sorting approach is used for each individual to create a Pareto rank, and a crowding distance assignment method is applied to implement density estimation. NSGA-II prefers the point with a lower rank value or the point located in a region with fewer points if both of the points belong to the same front. By combining a fast non-dominated sorting method, an elitism scheme and a parameter-less sharing method with its origin, NSGA-II is claimed to produce a better spread of solutions in some testing problems. The intense search ability on multi-objective problems motivated us to choose NSGA-II as the comparative algorithm.

In SPEA2, a fitness assignment scheme is used, which takes each individual into account how many individuals it dominates and it is dominated by; a nearest neighbor density estimation technique is incorporated, which allows more precise guidance of the search process; a new archive truncation method guarantees the preservation of boundary solutions. SPEA2 has been proved to perform well in convergence and diversity, so we use SPEA2 as a comparative algorithm.

MOTS is presented to solve a two-stage hybrid flow shop scheduling problem with SDST and preventive maintenance. Some parallel tabu lists and the Pareto dominance concept are used to generate non-nominated solutions in MOTS. MOTS can solve DTHFSP by deleting a code string corresponding to preventive maintenance and adding a code string corresponding to the factory. So, MOTS is used as another comparative algorithm.

Metric $\rho$ is the proportion of solutions that an algorithm can provide for the reference solution set. $\rho$ is calculated as

19 $$\begin{aligned} \rho =\frac{\left| \left\{ x \in \Omega _{A} \mid x \in \Omega ^{*}\right\} \right| }{\left| \Omega ^{*}\right| } \end{aligned}$$

where $\Omega _A$ is the non-dominated solutions obtained by algorithm *A* and $\Omega ^*$ represents the reference solution set.

Metric ${{\mathscr {C}}}$^74^ is applied to compare the approximate Pareto optimal set obtained by algorithms. ${{\mathscr {C}}}\left( {L,B} \right)$ measures the fraction of members of *B* that are dominated by members of *L*.

20 $$\begin{aligned} {{\mathscr {C}}}\left( {L,B} \right) = \frac{{\left| {\left\{ {b \in B:\exists h \in L,h \succ b} \right\} } \right| }}{{\left| B \right| }} \end{aligned}$$

where $x \succ y$ indicates that *x* dominates *y*, which is defined in Sect. 4.4.

Metric *IGD*^75,76^ is a comprehensive performance indicator to evaluate algorithms. The smaller the value of *IGS*, the better algorithm’s overall performance of *A*. *IGD* is calculated as

21 $$\begin{aligned} IGD\left( \Omega _{A}, \Omega ^{*}\right) =\frac{1}{\left| \Omega ^{*}\right| } \sum _{x \in \Omega ^{*}} \min _{y \in \Omega _{A}} d(x, y) \end{aligned}$$

where *d*(*x*, *y*) is the Euclidean distance between solution *x* and *y* by normalized objectives.

### Parameter settings

There are two parameters $max \_it$ and $\beta$ for CVNS. We used three settings of $0.05 \times n \times s$, $0.10 \times n \times s$ and $0.15 \times n \times s$ for $max\_it$ and 8000, 9000, 10,000 for $\beta$. There are 9 combinations for parameters. Based on the results measured using the above metrics, the best results are achieved using the following setting: $max\_it=0.10 \times n \times s$, $\beta =10{,}000$.

To apply NSGA-II, the above coding method and decoding procedure are adopted, eight neighborhood structures can be chosen as mutation, we applied five mutation operators using five neighborhood structures ${{\mathscr {N}}}_1,{{\mathscr {N}}}_2,{{\mathscr {N}}}_3,{{\mathscr {N}}}_5,{{\mathscr {N}}}_6$, because NSGA-II with these mutations can have the best performance. Two-point crossovers act on machine assignment string and scheduling string, respectively. SPEA2 is also implemented using the same way as NSGA-II. In these algorithms, five mutations are done sequentially, and so do two crossovers. In MOTS, only the operations for the factory string need to add. All parameters of NSGA-II, SPEA2 and MOTS are directly selected from Deb et al.^71^, Zitzler et al.^72^ and Wang and Liu^73^ except the stopping condition. For a fair comparison, the termination condition of all algorithms is $0.10 \times n \times s$. Experiments showed that parameter settings of three comparative algorithms could result in the best results in most instances, so these settings are used.

### Results and analyses

**Figure 4:**
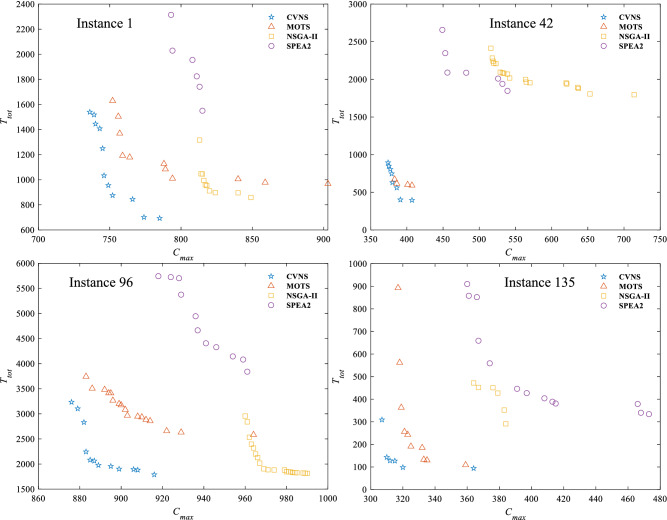
Distribution of non-dominated solutions of three algorithms.

**Figure 5:**
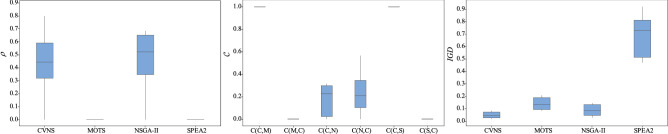
The box plots of three metrics for instances with 2 factories.

**Figure 6:**
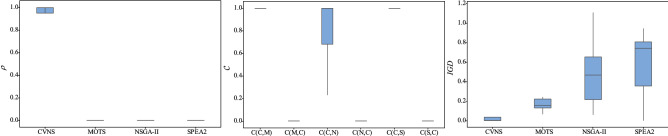
The box plots of three metrics for instances with 3 factories.

**Figure 7:**
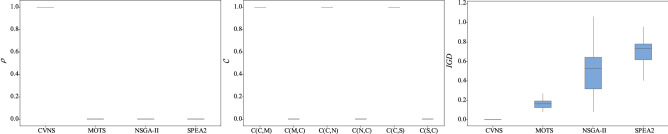
The box plots of three metrics for instances with 4 factories.

**Figure 8:**
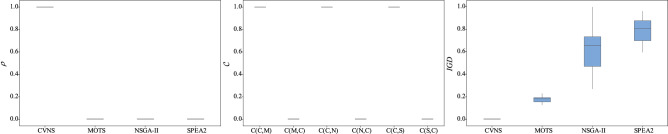
The box plots of three metrics for instances with 5 factories.

**Figure 9:**
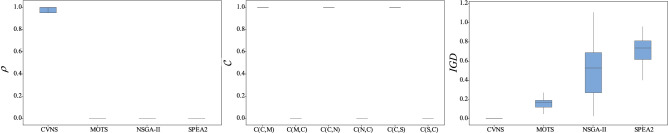
The box plots of three metrics for all instances.

Four algorithms randomly run 20 times for each instance. The computational results of four algorithms are reported in Tables 3, 4 and 5. The reference set $\Omega ^*$ is composed of non-dominated solutions obtained by four algorithms. Symbol ‘Ins’ indicates instance. Symbols ‘N’, ‘C’, ‘S’ and ‘M’ represent NSGA-II, CVNS, SPEA2 and MOTS. Figure 4 describes the distribution of non-dominated solutions on objective space of four algorithms. To make the results statistically convincing, the *p*-value results of paired-sample *t*-test are provided in Table 6. To show the overall distribution of all algorithms on all metrics, the box plot are provided in Figs. 5, 6, 7, 8 and 9.

**Table 3:** Computational results of four algorithms on metric $\rho$.

Ins	CVNS	MOTS	NSGA-II	SPEA2	Ins	CVNS	MOTS	NSGA-II	SPEA2	Ins	CVNS	MOTS	NSGA-II	SPEA2
1	1.000	0.000	0.000	0.000	64	1.000	0.000	0.000	0.000	127	1.000	0.000	0.000	0.000
2	0.667	0.000	0.333	0.000	65	1.000	0.000	0.000	0.000	128	1.000	0.000	0.000	0.000
3	1.000	0.000	0.000	0.000	66	1.000	0.000	0.000	0.000	129	1.000	0.000	0.000	0.000
4	0.758	0.000	0.242	0.000	67	1.000	0.000	0.000	0.000	130	1.000	0.000	0.000	0.000
5	0.457	0.000	0.543	0.000	68	1.000	0.000	0.000	0.000	131	1.000	0.000	0.000	0.000
6	0.706	0.000	0.294	0.000	69	1.000	0.000	0.000	0.000	132	1.000	0.000	0.000	0.000
7	0.821	0.000	0.179	0.000	70	1.000	0.000	0.000	0.000	133	1.000	0.000	0.000	0.000
8	0.429	0.000	0.571	0.000	71	1.000	0.000	0.000	0.000	134	1.000	0.000	0.000	0.000
9	0.431	0.000	0.569	0.000	72	1.000	0.000	0.000	0.000	135	1.000	0.000	0.000	0.000
10	0.476	0.000	0.524	0.000	73	1.000	0.000	0.000	0.000	136	0.000	0.000	0.000	1.000
11	1.000	0.000	0.000	0.000	74	1.000	0.000	0.000	0.000	137	1.000	0.000	0.000	0.000
12	1.000	0.000	0.000	0.000	75	1.000	0.000	0.000	0.000	138	1.000	0.000	0.000	0.000
13	1.000	0.000	0.000	0.000	76	1.000	0.000	0.000	0.000	139	1.000	0.000	0.000	0.000
14	1.000	0.000	0.000	0.000	77	1.000	0.000	0.000	0.000	140	1.000	0.000	0.000	0.000
15	1.000	0.000	0.000	0.000	78	1.000	0.000	0.000	0.000	141	1.000	0.000	0.000	0.000
16	1.000	0.000	0.000	0.000	79	1.000	0.000	0.000	0.000	142	1.000	0.000	0.000	0.000
17	1.000	0.000	0.000	0.000	80	1.000	0.000	0.000	0.000	143	1.000	0.000	0.000	0.000
18	1.000	0.000	0.000	0.000	81	1.000	0.000	0.000	0.000	144	1.000	0.000	0.000	0.000
19	1.000	0.000	0.000	0.000	82	1.000	0.000	0.000	0.000	145	0.800	0.200	0.000	0.000
20	1.000	0.000	0.000	0.000	83	1.000	0.000	0.000	0.000	146	1.000	0.000	0.000	0.000
21	0.875	0.125	0.000	0.000	84	1.000	0.000	0.000	0.000	147	1.000	0.000	0.000	0.000
22	1.000	0.000	0.000	0.000	85	1.000	0.000	0.000	0.000	148	1.000	0.000	0.000	0.000
23	0.909	0.091	0.000	0.000	86	1.000	0.000	0.000	0.000	149	1.000	0.000	0.000	0.000
24	1.000	0.000	0.000	0.000	87	1.000	0.000	0.000	0.000	150	1.000	0.000	0.000	0.000
25	1.000	0.000	0.000	0.000	88	1.000	0.000	0.000	0.000	151	1.000	0.000	0.000	0.000
26	1.000	0.000	0.000	0.000	89	1.000	0.000	0.000	0.000	152	1.000	0.000	0.000	0.000
27	1.000	0.000	0.000	0.000	90	1.000	0.000	0.000	0.000	153	1.000	0.000	0.000	0.000
28	0.679	0.000	0.321	0.000	91	1.000	0.000	0.000	0.000	154	1.000	0.000	0.000	0.000
29	1.000	0.000	0.000	0.000	92	1.000	0.000	0.000	0.000	155	1.000	0.000	0.000	0.000
30	1.000	0.000	0.000	0.000	93	1.000	0.000	0.000	0.000	156	1.000	0.000	0.000	0.000
31	1.000	0.000	0.000	0.000	94	1.000	0.000	0.000	0.000	157	1.000	0.000	0.000	0.000
32	1.000	0.000	0.000	0.000	95	1.000	0.000	0.000	0.000	158	1.000	0.000	0.000	0.000
33	1.000	0.000	0.000	0.000	96	1.000	0.000	0.000	0.000	159	1.000	0.000	0.000	0.000
34	1.000	0.000	0.000	0.000	97	0.708	0.000	0.292	0.000	160	1.000	0.000	0.000	0.000
35	1.000	0.000	0.000	0.000	98	0.513	0.000	0.487	0.000	161	1.000	0.000	0.000	0.000
36	1.000	0.000	0.000	0.000	99	0.737	0.000	0.263	0.000	162	1.000	0.000	0.000	0.000
37	1.000	0.000	0.000	0.000	100	0.720	0.000	0.280	0.000	163	1.000	0.000	0.000	0.000
38	1.000	0.000	0.000	0.000	101	0.455	0.000	0.545	0.000	164	1.000	0.000	0.000	0.000
39	1.000	0.000	0.000	0.000	102	0.450	0.000	0.550	0.000	165	1.000	0.000	0.000	0.000
40	1.000	0.000	0.000	0.000	103	0.339	0.000	0.661	0.000	166	1.000	0.000	0.000	0.000
41	1.000	0.000	0.000	0.000	104	0.429	0.000	0.571	0.000	167	1.000	0.000	0.000	0.000
42	1.000	0.000	0.000	0.000	105	1.000	0.000	0.000	0.000	168	1.000	0.000	0.000	0.000
43	1.000	0.000	0.000	0.000	106	1.000	0.000	0.000	0.000	169	1.000	0.000	0.000	0.000
44	1.000	0.000	0.000	0.000	107	1.000	0.000	0.000	0.000	170	1.000	0.000	0.000	0.000
45	1.000	0.000	0.000	0.000	108	1.000	0.000	0.000	0.000	171	1.000	0.000	0.000	0.000
46	1.000	0.000	0.000	0.000	109	1.000	0.000	0.000	0.000	172	1.000	0.000	0.000	0.000
47	1.000	0.000	0.000	0.000	110	1.000	0.000	0.000	0.000	173	1.000	0.000	0.000	0.000
48	1.000	0.000	0.000	0.000	111	1.000	0.000	0.000	0.000	174	1.000	0.000	0.000	0.000
49	1.000	0.000	0.000	0.000	112	1.000	0.000	0.000	0.000	175	1.000	0.000	0.000	0.000
50	1.000	0.000	0.000	0.000	113	1.000	0.000	0.000	0.000	176	1.000	0.000	0.000	0.000
51	0.500	0.500	0.000	0.000	114	1.000	0.000	0.000	0.000	177	1.000	0.000	0.000	0.000
52	1.000	0.000	0.000	0.000	115	1.000	0.000	0.000	0.000	178	1.000	0.000	0.000	0.000
53	0.000	0.000	0.000	1.000	116	1.000	0.000	0.000	0.000	179	1.000	0.000	0.000	0.000
54	1.000	0.000	0.000	0.000	117	1.000	0.000	0.000	0.000	180	1.000	0.000	0.000	0.000
55	1.000	0.000	0.000	0.000	118	1.000	0.000	0.000	0.000	181	1.000	0.000	0.000	0.000
56	1.000	0.000	0.000	0.000	119	0.000	0.000	0.000	1.000	182	1.000	0.000	0.000	0.000
57	1.000	0.000	0.000	0.000	120	0.810	0.190	0.000	0.000	183	1.000	0.000	0.000	0.000
58	1.000	0.000	0.000	0.000	121	1.000	0.000	0.000	0.000	184	1.000	0.000	0.000	0.000
59	1.000	0.000	0.000	0.000	122	1.000	0.000	0.000	0.000	185	1.000	0.000	0.000	0.000
60	1.000	0.000	0.000	0.000	123	1.000	0.000	0.000	0.000	186	1.000	0.000	0.000	0.000
61	1.000	0.000	0.000	0.000	124	0.690	0.000	0.310	0.000	187	1.000	0.000	0.000	0.000
62	1.000	0.000	0.000	0.000	125	1.000	0.000	0.000	0.000	188	1.000	0.000	0.000	0.000
63	1.000	0.000	0.000	0.000	126	1.000	0.000	0.000	0.000

**Table 4:** Computational results of four algorithms on metric ${ \mathscr {C}}$.

Ins	${{\mathscr {C}}}(C,M)$	${{\mathscr {C}}}(M,C)$	${{\mathscr {C}}}(C,N)$	${{\mathscr {C}}}(N,C)$	${{\mathscr {C}}}(C,S)$	${{\mathscr {C}}}(S,C)$	Ins	${\mathscr {C}}(C,M)$	${{\mathscr {C}}}(M,C)$	${{\mathscr {C}}}(C,N)$	${{\mathscr {C}}}(N,C)$	${\mathscr {C}}(C,S)$	${{\mathscr {C}}}(S,C)$	Ins	${{\mathscr {C}}}(C,M)$	${{\mathscr {C}}}(M,C)$	${{\mathscr {C}}}(C,N)$	${{\mathscr {C}}}(N,C)$	${{\mathscr {C}}}(C,S)$	${{\mathscr {C}}}(S,C)$
1	1.000	0.000	1.000	0.000	1.000	0.000	64	1.000	0.000	1.000	0.000	1.000	0.000	127	1.000	0.000	1.000	0.000	1.000	0.000
2	1.000	0.000	0.714	0.000	1.000	0.000	65	1.000	0.000	1.000	0.000	1.000	0.000	128	1.000	0.000	1.000	0.000	1.000	0.000
3	1.000	0.000	1.000	0.000	1.000	0.000	66	1.000	0.000	1.000	0.000	1.000	0.000	129	1.000	0.000	1.000	0.000	1.000	0.000
4	1.000	0.000	0.765	0.000	1.000	0.000	67	1.000	0.000	1.000	0.000	1.000	0.000	130	1.000	0.000	1.000	0.000	1.000	0.000
5	1.000	0.000	0.578	0.000	1.000	0.000	68	1.000	0.000	1.000	0.000	1.000	0.000	131	1.000	0.000	1.000	0.000	1.000	0.000
6	1.000	0.000	0.714	0.000	1.000	0.000	69	1.000	0.000	1.000	0.000	1.000	0.000	132	1.000	0.000	1.000	0.000	1.000	0.000
7	1.000	0.000	0.688	0.000	1.000	0.000	70	1.000	0.000	1.000	0.000	1.000	0.000	133	1.000	0.000	1.000	0.000	1.000	0.000
8	1.000	0.000	0.143	0.000	1.000	0.000	71	1.000	0.000	1.000	0.000	1.000	0.000	134	1.000	0.000	1.000	0.000	1.000	0.000
9	1.000	0.000	0.411	0.000	1.000	0.000	72	1.000	0.000	1.000	0.000	1.000	0.000	135	1.000	0.000	1.000	0.000	1.000	0.000
10	1.000	0.000	0.043	0.000	1.000	0.000	73	1.000	0.000	1.000	0.000	1.000	0.000	136	1.000	0.000	1.000	0.000	0.000	1.000
11	1.000	0.000	1.000	0.000	1.000	0.000	74	1.000	0.000	1.000	0.000	1.000	0.000	137	1.000	0.000	1.000	0.000	1.000	0.000
12	1.000	0.000	1.000	0.000	1.000	0.000	75	1.000	0.000	1.000	0.000	1.000	0.000	138	1.000	0.000	1.000	0.000	1.000	0.000
13	1.000	0.000	1.000	0.000	1.000	0.000	76	1.000	0.000	1.000	0.000	1.000	0.000	139	1.000	0.000	1.000	0.000	1.000	0.000
14	1.000	0.000	1.000	0.000	1.000	0.000	77	1.000	0.000	1.000	0.000	1.000	0.000	140	1.000	0.000	1.000	0.000	1.000	0.000
15	1.000	0.000	1.000	0.000	1.000	0.000	78	1.000	0.000	1.000	0.000	1.000	0.000	141	1.000	0.000	1.000	0.000	1.000	0.000
16	1.000	0.000	1.000	0.000	1.000	0.000	79	1.000	0.000	1.000	0.000	1.000	0.000	142	1.000	0.000	1.000	0.000	1.000	0.000
17	1.000	0.000	1.000	0.000	1.000	0.000	80	1.000	0.000	1.000	0.000	1.000	0.000	143	1.000	0.000	1.000	0.000	1.000	0.000
18	1.000	0.000	1.000	0.000	1.000	0.000	81	1.000	0.000	1.000	0.000	1.000	0.000	144	1.000	0.000	1.000	0.000	1.000	0.000
19	1.000	0.000	1.000	0.000	1.000	0.000	82	1.000	0.000	1.000	0.000	1.000	0.000	145	0.875	0.000	1.000	0.000	1.000	0.000
20	1.000	0.000	1.000	0.000	1.000	0.000	83	1.000	0.000	1.000	0.000	1.000	0.000	146	1.000	0.000	1.000	0.000	1.000	0.000
21	0.875	0.000	1.000	0.000	1.000	0.000	84	1.000	0.000	1.000	0.000	1.000	0.000	147	1.000	0.000	1.000	0.000	1.000	0.000
22	1.000	0.000	1.000	0.000	1.000	0.000	85	1.000	0.000	1.000	0.000	1.000	0.000	148	1.000	0.000	1.000	0.000	1.000	0.000
23	0.889	0.000	1.000	0.000	1.000	0.000	86	1.000	0.000	1.000	0.000	1.000	0.000	149	1.000	0.000	1.000	0.000	1.000	0.000
24	1.000	0.000	1.000	0.000	1.000	0.000	87	1.000	0.000	1.000	0.000	1.000	0.000	150	1.000	0.000	1.000	0.000	1.000	0.000
25	1.000	0.000	1.000	0.000	1.000	0.000	88	1.000	0.000	1.000	0.000	1.000	0.000	151	1.000	0.000	1.000	0.000	1.000	0.000
26	1.000	0.000	1.000	0.000	1.000	0.000	89	1.000	0.000	1.000	0.000	1.000	0.000	152	1.000	0.000	1.000	0.000	1.000	0.000
27	1.000	0.000	1.000	0.000	1.000	0.000	90	1.000	0.000	1.000	0.000	1.000	0.000	153	1.000	0.000	1.000	0.000	1.000	0.000
28	1.000	0.000	0.400	0.000	1.000	0.000	91	1.000	0.000	1.000	0.000	1.000	0.000	154	1.000	0.000	1.000	0.000	1.000	0.000
29	1.000	0.000	1.000	0.000	1.000	0.000	92	1.000	0.000	1.000	0.000	1.000	0.000	155	1.000	0.000	1.000	0.000	1.000	0.000
30	1.000	0.000	1.000	0.000	1.000	0.000	93	1.000	0.000	1.000	0.000	1.000	0.000	156	1.000	0.000	1.000	0.000	1.000	0.000
31	1.000	0.000	1.000	0.000	1.000	0.000	94	1.000	0.000	1.000	0.000	1.000	0.000	157	1.000	0.000	1.000	0.000	1.000	0.000
32	1.000	0.000	1.000	0.000	1.000	0.000	95	1.000	0.000	1.000	0.000	1.000	0.000	158	1.000	0.000	1.000	0.000	1.000	0.000
33	1.000	0.000	1.000	0.000	1.000	0.000	96	1.000	0.000	1.000	0.000	1.000	0.000	159	1.000	0.000	1.000	0.000	1.000	0.000
34	1.000	0.000	1.000	0.000	1.000	0.000	97	1.000	0.000	0.750	0.000	1.000	0.000	160	1.000	0.000	1.000	0.000	1.000	0.000
35	1.000	0.000	1.000	0.000	1.000	0.000	98	1.000	0.000	0.345	0.000	1.000	0.000	161	1.000	0.000	1.000	0.000	1.000	0.000
36	1.000	0.000	1.000	0.000	1.000	0.000	99	1.000	0.000	0.815	0.000	1.000	0.000	162	1.000	0.000	1.000	0.000	1.000	0.000
37	1.000	0.000	1.000	0.000	1.000	0.000	100	1.000	0.000	0.854	0.000	1.000	0.000	163	1.000	0.000	1.000	0.000	1.000	0.000
38	1.000	0.000	1.000	0.000	1.000	0.000	101	1.000	0.000	0.333	0.000	1.000	0.000	164	1.000	0.000	1.000	0.000	1.000	0.000
39	1.000	0.000	1.000	0.000	1.000	0.000	102	1.000	0.000	0.353	0.000	1.000	0.000	165	1.000	0.000	1.000	0.000	1.000	0.000
40	1.000	0.000	1.000	0.000	1.000	0.000	103	1.000	0.000	0.093	0.000	1.000	0.000	166	1.000	0.000	1.000	0.000	1.000	0.000
41	1.000	0.000	1.000	0.000	1.000	0.000	104	1.000	0.000	0.000	0.000	1.000	0.000	167	1.000	0.000	1.000	0.000	1.000	0.000
42	1.000	0.000	1.000	0.000	1.000	0.000	105	1.000	0.000	1.000	0.000	1.000	0.000	168	1.000	0.000	1.000	0.000	1.000	0.000
43	1.000	0.000	1.000	0.000	1.000	0.000	106	1.000	0.000	1.000	0.000	1.000	0.000	169	1.000	0.000	1.000	0.000	1.000	0.000
44	1.000	0.000	1.000	0.000	1.000	0.000	107	1.000	0.000	1.000	0.000	1.000	0.000	170	1.000	0.000	1.000	0.000	1.000	0.000
45	1.000	0.000	1.000	0.000	1.000	0.000	108	1.000	0.000	1.000	0.000	1.000	0.000	171	1.000	0.000	1.000	0.000	1.000	0.000
46	1.000	0.000	1.000	0.000	1.000	0.000	109	1.000	0.000	1.000	0.000	1.000	0.000	172	1.000	0.000	1.000	0.000	1.000	0.000
47	1.000	0.000	1.000	0.000	1.000	0.000	110	1.000	0.000	1.000	0.000	1.000	0.000	173	1.000	0.000	1.000	0.000	1.000	0.000
48	1.000	0.000	1.000	0.000	1.000	0.000	111	1.000	0.000	1.000	0.000	1.000	0.000	174	1.000	0.000	1.000	0.000	1.000	0.000
49	1.000	0.000	1.000	0.000	1.000	0.000	112	1.000	0.000	1.000	0.000	1.000	0.000	175	1.000	0.000	1.000	0.000	1.000	0.000
50	1.000	0.000	1.000	0.000	1.000	0.000	113	1.000	0.000	1.000	0.000	1.000	0.000	176	1.000	0.000	1.000	0.000	1.000	0.000
51	0.571	0.000	1.000	0.000	1.000	0.000	114	1.000	0.000	1.000	0.000	1.000	0.000	177	1.000	0.000	1.000	0.000	1.000	0.000
52	1.000	0.000	1.000	0.000	1.000	0.000	115	1.000	0.000	1.000	0.000	1.000	0.000	178	1.000	0.000	1.000	0.000	1.000	0.000
53	1.000	0.000	1.000	0.000	0.000	1.000	116	1.000	0.000	1.000	0.000	1.000	0.000	179	1.000	0.000	1.000	0.000	1.000	0.000
54	1.000	0.000	1.000	0.000	1.000	0.000	117	1.000	0.000	1.000	0.000	1.000	0.000	180	1.000	0.000	1.000	0.000	1.000	0.000
55	1.000	0.000	1.000	0.000	1.000	0.000	118	1.000	0.000	1.000	0.000	1.000	0.000	181	1.000	0.000	1.000	0.000	1.000	0.000
56	1.000	0.000	1.000	0.000	1.000	0.000	119	1.000	0.000	1.000	0.000	0.000	1.000	182	1.000	0.000	1.000	0.000	1.000	0.000
57	1.000	0.000	1.000	0.000	1.000	0.000	120	0.692	0.105	1.000	0.000	1.000	0.000	183	1.000	0.000	1.000	0.000	1.000	0.000
58	1.000	0.000	1.000	0.000	1.000	0.000	121	1.000	0.000	1.000	0.000	1.000	0.000	184	1.000	0.000	1.000	0.000	1.000	0.000
59	1.000	0.000	1.000	0.000	1.000	0.000	122	1.000	0.000	1.000	0.000	1.000	0.000	185	1.000	0.000	1.000	0.000	1.000	0.000
60	1.000	0.000	1.000	0.000	1.000	0.000	123	1.000	0.000	1.000	0.000	1.000	0.000	186	1.000	0.000	1.000	0.000	1.000	0.000
61	1.000	0.000	1.000	0.000	1.000	0.000	124	1.000	0.000	0.870	0.000	1.000	0.000	187	1.000	0.000	1.000	0.000	1.000	0.000
62	1.000	0.000	1.000	0.000	1.000	0.000	125	1.000	0.000	1.000	0.000	1.000	0.000	188	1.000	0.000	1.000	0.000	1.000	0.000
63	1.000	0.000	1.000	0.000	1.000	0.000	126	1.000	0.000	1.000	0.000	1.000	0.000

**Table 5:** Computational results of four algorithms on metric *IGD*.

Ins	CVNS	MOTS	NSGA-II	SPEA2	Ins	CVNS	MOTS	NSGA-II	SPEA2	Ins	CVNS	MOTS	NSGA-II	SPEA2
1	0.000	0.146	0.388	0.505	64	0.000	0.081	0.938	1.106	127	0.000	0.155	1.081	1.024
2	0.219	0.352	0.408	0.538	65	0.000	0.049	1.014	0.862	128	0.000	0.113	0.944	0.975
3	0.000	0.216	0.762	0.848	66	0.000	0.130	0.842	1.142	129	0.000	0.118	1.105	0.842
4	0.083	0.230	0.444	0.727	67	0.000	0.091	1.119	1.127	130	0.000	0.136	0.779	1.182
5	0.451	0.425	0.375	0.890	68	0.000	0.117	1.183	1.110	131	0.000	0.138	0.932	1.209
6	0.123	0.163	0.325	0.818	69	0.000	0.092	1.132	1.199	132	0.000	0.201	1.043	1.134
7	0.132	0.190	0.736	1.011	70	0.000	0.135	1.074	1.290	133	0.000	0.082	0.944	1.125
8	0.330	0.173	0.282	0.907	71	0.000	0.180	1.037	0.800	134	0.000	0.146	0.967	1.096
9	0.370	0.485	0.287	0.830	72	0.000	0.088	1.016	1.039	135	0.000	0.090	0.423	0.566
10	0.333	0.371	0.329	0.924	73	0.000	0.178	1.082	1.074	136	0.700	0.760	1.140	0.000
11	0.000	0.129	0.465	0.274	74	0.000	0.077	0.987	0.911	137	0.000	0.041	0.740	0.908
12	0.000	0.161	0.825	0.564	75	0.000	0.121	0.843	1.041	138	0.000	0.102	0.538	0.768
13	0.000	0.099	0.970	0.950	76	0.000	0.116	1.030	1.158	139	0.000	0.086	0.670	0.888
14	0.000	0.097	0.901	0.720	77	0.000	0.147	0.951	1.108	140	0.000	0.134	0.594	0.872
15	0.000	0.092	0.796	1.052	78	0.000	0.092	0.688	1.089	141	0.000	0.133	0.560	0.612
16	0.000	0.128	0.954	0.949	79	0.000	0.128	1.106	0.943	142	0.000	0.140	0.681	1.130
17	0.000	0.152	0.945	1.158	80	0.000	0.054	1.008	1.000	143	0.000	0.111	0.660	1.105
18	0.000	0.110	1.048	1.094	81	0.000	0.054	0.814	1.038	144	0.000	0.155	0.852	1.239
19	0.000	0.101	0.881	1.116	82	0.000	0.066	0.984	1.015	145	0.006	0.047	0.678	0.421
20	0.000	0.111	0.665	1.110	83	0.000	0.095	0.934	1.038	146	0.000	0.134	1.040	0.775
21	0.009	0.078	0.826	0.320	84	0.000	0.132	1.138	1.194	147	0.000	0.243	0.907	1.016
22	0.000	0.141	0.670	0.796	85	0.000	0.082	1.109	1.067	148	0.000	0.121	1.069	0.792
23	0.036	0.036	0.657	0.956	86	0.000	0.126	0.877	1.140	149	0.000	0.175	0.838	1.041
24	0.000	0.114	0.934	0.894	87	0.000	0.117	1.009	0.997	150	0.000	0.166	0.489	1.053
25	0.000	0.153	0.944	0.928	88	0.000	0.142	1.202	1.056	151	0.000	0.096	0.702	0.980
26	0.000	0.158	0.835	1.107	89	0.000	0.091	1.235	0.967	152	0.000	0.123	0.909	1.130
27	0.000	0.062	0.699	0.974	90	0.000	0.066	0.914	1.264	153	0.000	0.143	0.697	1.015
28	0.219	0.318	0.539	0.975	91	0.000	0.145	1.160	1.125	154	0.000	0.128	0.763	1.122
29	0.000	0.164	0.867	1.149	92	0.000	0.129	1.121	1.297	155	0.000	0.212	1.294	0.840
30	0.000	0.102	0.676	1.028	93	0.000	0.097	1.154	1.171	156	0.000	0.150	1.121	0.533
31	0.000	0.387	1.139	0.506	94	0.000	0.169	1.179	1.226	157	0.000	0.198	1.093	1.011
32	0.000	0.244	1.119	0.832	95	0.000	0.037	0.325	0.429	158	0.000	0.150	1.179	0.984
33	0.000	0.145	0.609	0.840	96	0.000	0.225	0.623	0.705	159	0.000	0.164	1.197	1.038
34	0.000	0.106	0.974	0.913	97	0.161	0.210	0.207	0.625	160	0.000	0.095	1.210	0.911
35	0.000	0.120	0.915	1.141	98	0.133	0.175	0.196	0.767	161	0.000	0.127	1.077	1.231
36	0.000	0.130	0.995	0.910	99	0.157	0.204	0.604	0.864	162	0.000	0.170	1.247	1.331
37	0.000	0.121	0.823	1.125	100	0.191	0.341	0.386	0.878	163	0.000	0.119	1.027	1.262
38	0.000	0.092	0.798	1.026	101	0.347	0.361	0.283	0.753	164	0.000	0.119	0.988	1.146
39	0.000	0.127	1.015	1.184	102	0.332	0.428	0.307	0.780	165	0.000	0.186	0.837	0.858
40	0.000	0.099	0.887	1.216	103	0.417	0.456	0.224	0.887	166	0.000	0.190	1.103	0.806
41	0.000	0.134	0.845	0.387	104	0.308	0.438	0.306	0.986	167	0.000	0.172	0.797	1.056
42	0.000	0.063	0.763	0.667	105	0.000	0.349	0.810	0.700	168	0.000	0.104	0.763	0.950
43	0.000	0.116	0.956	0.723	106	0.000	0.229	0.816	0.714	169	0.000	0.179	0.870	1.031
44	0.000	0.061	0.536	0.753	107	0.000	0.236	0.912	0.936	170	0.000	0.150	0.756	1.060
45	0.000	0.114	0.822	0.907	108	0.000	0.172	0.801	0.989	171	0.000	0.145	0.941	1.027
46	0.000	0.090	0.609	0.929	109	0.000	0.124	1.014	0.996	172	0.000	0.197	0.634	0.978
47	0.000	0.100	0.726	0.901	110	0.000	0.153	0.962	0.966	173	0.000	0.128	1.110	0.682
48	0.000	0.102	0.549	0.878	111	0.000	0.134	0.973	1.093	174	0.000	0.196	1.108	1.090
49	0.000	0.105	0.632	0.976	112	0.000	0.188	0.941	1.218	175	0.000	0.193	1.224	1.172
50	0.000	0.172	0.568	1.178	113	0.000	0.107	0.803	1.054	176	0.000	0.131	1.282	1.033
51	0.067	0.070	0.966	0.341	114	0.000	0.171	1.071	1.315	177	0.000	0.176	1.110	1.089
52	0.000	0.202	1.025	0.807	115	0.000	0.126	0.689	0.338	178	0.000	0.166	1.169	1.112
53	0.426	0.461	0.981	0.000	116	0.000	0.090	0.819	0.631	179	0.000	0.095	0.689	1.013
54	0.000	0.125	0.696	0.920	117	0.000	0.138	0.618	0.838	180	0.000	0.160	0.976	1.131
55	0.000	0.136	0.874	1.122	118	0.000	0.081	0.521	0.754	181	0.000	0.159	1.114	1.141
56	0.000	0.189	1.045	1.058	119	0.739	0.793	1.214	0.000	182	0.000	0.118	1.202	1.250
57	0.000	0.059	0.971	1.092	120	0.004	0.083	0.563	0.964	183	0.000	0.128	1.104	0.982
58	0.000	0.100	0.851	1.043	121	0.000	0.156	0.698	1.131	184	0.000	0.160	1.125	1.054
59	0.000	0.091	0.743	0.965	122	0.000	0.090	0.629	1.144	185	0.000	0.188	1.320	1.299
60	0.000	0.148	0.672	1.100	123	0.000	0.109	0.758	1.014	186	0.000	0.143	1.066	1.305
61	0.000	0.228	1.139	0.360	124	0.223	0.281	0.416	1.084	187	0.000	0.148	0.943	1.132
62	0.000	0.137	1.323	0.722	125	0.000	0.221	1.073	0.480	188	0.000	0.147	1.157	1.117
63	0.000	0.084	1.054	0.948	126	0.000	0.231	0.954	0.923

**Table 6:** Results of paired-sample *t*-test.

*t*-test	*p*-value($\rho$)	*p*-value($\mathscr {C}$)	*p*-value(*IGD*)
*t*-test(CVNS,MOTS)	0.00	0.00	0.00
*t*-test(CVNS,NSGA-II)	0.00	0.00	0.00
*t*-test(CVNS,SPEA2)	0.00	0.00	0.00

#### Results analyses

As shown in Table 3, CVNS provides a larger proportion of solutions to the reference solution set than the three comparison algorithms in most instances. In 71 instances, the $\rho$ of CVNS equals 1, which means that all solutions of the reference set are provided by CVNS. As can be seen from Figs. 5, 6, 7, 8 and 9, only when $F=2$, the performance of NSGA-II is comparable to that of CVNS; when $F=3,4,5$, CVNS is significantly better than that of NSGA-II. It is noted that when $F=2,3,4,5$, CNVS performed better than SPEA2 and MOTS. So CNVS can provide more non-dominated solutions than comparison algorithms.

As stated in Table 4, CVNS performed better than its comparison algorithms regarding dominance ratio. In most instances, $\mathscr {C}$ of CVNS equals 1, and $\mathscr {C}$ of its comparison algorithms equals 0, that is, solutions of CVNS dominate all solutions obtained by the comparison algorithm. CVNS can get higher quality calculation results under different factories, especially when the number of factories increases. Therefore, CNVS is superior in the dominance relationship compared with comparison algorithms.

As exhibited in Table 5, CVNS converges better than NSGA-II, SPEA2 and MOTS in most instances. *IGD* of CVNS is less than that of three comparison algorithms on 176 instances and equal to 0 on 163 instances; that is, all members of the reference set $\Omega ^*$ are generated by CVNS. So CVNS has better convergence.

#### Robustness analysis

In all 188 instances, the relevant variables are the range of processing time, the range of due date, the number of jobs and the number of parallel machines. As can be seen from the computational results of the three metrics in Tables 3, 4 and 5, CVNS is better than the comparison algorithms in most instances as these variables change, so CVNS has good robustness.

#### Sensitivity analysis

Instances 1–94 have a different range of processing time with instances 95–188. After the range of processing time is changed, CVNS can still obtain better results than the three comparison algorithms, as shown in Table 3, 4 and 5. In addition, as the number of factories changes, CVNS still outperforms comparison algorithms. As shown in the box plots of Figs. 5, 6, 7, 8 and 9, with the different factories, the metric $\rho$ tends to 1, the metric $\mathscr {C}$ tends to 1, and the metric *IGD* tends to 0; that is, CVNS performs better in different instances with the different number of factories.

The paired-sample *t*-test is a simple statistical test to testify to the performance of algorithms. The term t-test (A, B) means that paired t-test is conducted to judge whether algorithm A gives a better sample mean than B. We assume a significance level of 0.05. There is a significant difference between A and B in the statistical sense if the p-value is less than 0.05. As stated in the statistical results of Table 6, *p*-value($\rho$), *p*-value($\mathscr {C}$) and *p*-value(*IGD*) are all equal to 0, thus, CVNS has promising advantages on solving DTHFSP statistically. The same conclusion also can be drawn from Fig. 4.

The excellent performance of CVNS mainly results from its two cooperation mechanisms: the collaboration of global search and neighborhood search and the cooperation of two VNS algorithms; on the contrary, two GAs have strong global search ability and often low efficiency in local search, based on the above discussions, it can be concluded that CVNS is a promising method for solving bi-objective DTHFSP with SDST.

## Conclusions

Two-stage HFSP has been extensively investigated in the past decade; however, this problem is not studied in the multi-factory production network. This paper aims to solve DTHFSP with SDST by using a new algorithm called CVNS to minimize simultaneously total tardiness and makespan. DTHFSP is simplified by incorporating factory assignment into machine assignment of the first stage, and its solution is newly represented with a machine assignment string and a scheduling string. CVNS consists of two cooperated VNS algorithms, and in each VNS, neighborhood structures and global search have collaborated. Some neighborhood structures and global search operators are applied to produce new solutions. The current solution is periodically replaced with a member of the archive farthest from it. The performance of CVNS is tested by using 94 instances. The computational results show that CVNS is a promising method to solve the considered DTHFSP.

In the near future, we will continue to pay attention to distributed scheduling problems in the two-stage hybrid flow shop or the hybrid flow shop and try to solve the problem using meta-heuristics such as the imperialist competitive algorithm. We also deal with the above problems with energy-related objectives. Energy-efficient HFSP is also our future topic.

## Data Availability

All data generated or analysed during this study are included in this published article.
